# Long-term results after limited macular translocation surgery for wet age-related macular degeneration

**DOI:** 10.1371/journal.pone.0177241

**Published:** 2017-05-23

**Authors:** Hisaaki Oshima, Takeshi Iwase, Kohei Ishikawa, Kentaro Yamamoto, Hiroko Terasaki

**Affiliations:** Department of Ophthalmology, Nagoya University Graduate School of Medicine, Nagoya, Aichi, Japan; Massachusetts Eye & Ear Infirmary, Harvard Medical School, UNITED STATES

## Abstract

**Purpose:**

To evaluate the long-term results of limited macular translocation (LMT) surgery with radial chorioscleral outfolding in patients with wet age-related macular degeneration (AMD) and subfoveal choroidal neovascularization (CNV). In addition, to identify the factors associated with the final best-corrected visual acuity (BCVA).

**Methods:**

The medical records of 20 eyes of 20 consecutive patients (65.2±9.8 years) who had undergone LMT for the treatment of wet AMD and were followed for at least 5 years, were reviewed. The surgical outcomes including the BCVA, degree of foveal displacement, and complications were recorded.

**Results:**

The mean foveal displacement was 1332 ± 393 μm after the LMT. The CNV was removed in 16 eyes and photocoagulated in 4 eyes. The mean preoperative VA was 0.83 ± 0.33 logMAR units which significantly improved to 0.59 ± 0.37 logMAR units at 1 year after the surgery (*P* = 0.015). This BCVA was maintained at 0.59 ± 0.41 logMAR units on the final examination. The final BCVA was significantly correlated with that at 1 year after the surgery (r = 0.83, *P*<0.001). Multiple linear regression analysis showed that the final BCVA was significantly correlated with the BCVA at 1 year after the surgery (*P*<0.001), a recurrence of a CNV (*P* = 0.001), and the age (*P* = 0.022).

**Conclusions:**

LMT improves the BCVA significantly at 1 year, and the improved BCVA lasted for at least 5 years. These results indicate that the impaired function of the sensory retina at the fovea can recover on the new RPE after the displacement for at least 5 years. The ability to maintain good retinal function on the new RPE for a long period is important for future treatments of CNVs such as the transplantation of RPE cells and stem cells.

## Introduction

Age-related macular degeneration (AMD) remains a leading cause of legal blindness in adults 65-years and older.[1] The development of choroidal neovascularization (CNV) in eyes with wet AMD usually leads to a severe decrease of vision. Current medical treatments are aimed at preventing the progression of the AMD by treating the CNV membranes with intravitreal injections of anti-vascular endothelial growth factor (anti-VEGF),[2] triamcinolone,[3] photodynamic therapy,[4] or laser photocoagulation.[5] However, if the retinal pigment epithelium (RPE) underlying the fovea is damaged severely, the medical treatments have limited effects on the best-corrected visual acuity (BCVA).

To overcome this problem, new therapies such as surgical treatments have been performed and transplantation of RPE cells and stem cells are being tried.[6–8] Different surgical treatments have been developed for the advanced AMD lesions that were not appropriate for medical treatments. Macular translocation is performed to move the macula from the underlying damaged RPE to an area of healthier RPE.[9–11] Two surgical techniques of macular translocation have been used; full macular translocation with 360-degree retinotomy,[10–12] and limited macular translocation (LMT) with less extensive movement of the retina.[13–16] Each has advantages and disadvantages with respect to its effectiveness and complications. LMT has the advantage of being less invasive and having a lower rate of complications. On the other hand, its disadvantages include smaller and less predictable foveal displacement and development of foveal folds.[17] Although many studies have reported favorable outcomes, the longest follow-up period has been 2 years after the LMT.[14–20] The long-term results of LMT should provide important evidence on whether an impaired sensory retina can recover after it is moved onto healthy RPE, and the good visual function can be maintained for a longer period. A search of Medline did not extract any publications describing the outcomes of LMT after 2 years.

Thus, the purpose of this study was to investigate the outcomes of LMT surgery on the BCVA 5 or more years after the LMT in eyes with wet AMD. In addition, to identify the factors associated with the improved BCVA.

## Patients and methods

### Ethics statement

This was a retrospective, observational, comparative, single-center study, and the procedures were approved by the Institutional Review Board and the Ethics Committee of the Nagoya University Graduate School of Medicine. The procedures also conformed to the tenets of the Declaration of Helsinki. A written informed consent had been obtained from all of the patients for the surgery after an explanation of the procedures to be performed and possible complications. Verbal permission was also obtained to use the data collected for future research

### Subjects

We reviewed the medical records of all patients who had undergone LMT with diagonal chorioscleral outfolding for subfoveal wet AMD at the Nagoya University Hospital between July 2001 and November 2003. The surgical inclusion criteria were a BCVA worse than 20/40, CNV had not extended more than 1 disk diameter inferior to the center of the fovea, and no previous photocoagulation. Eyes with a follow-up period of less than 5 years were excluded.

The ophthalmic examinations consisted of measurements of the best-correct visual acuity (BCVA) and intraocular pressure, slit-lamp biomicroscopy, ophthalmoscopy, fundus photography, and fluorescein and indocyanine-green angiography. These examinations were performed before and at different times after the surgery. Surgical complications and side effects, such as a recurrence of the CNV, hemorrhage, macular hole (MH), and other complications were also recorded. The CNV size, the distance of the foveal displacement, and disc diameter were measured on the color fundus photographs and fluorescein angiograms. The size of the CNV, and the distance of the foveal displacement were converted to actual distance, assuming a vertical disc diameter of 1.88 mm. [18] [21] The Stratus^®^ OCT (OCT 3000TM, Carl Zeiss Meditec, Dublin, CA) and the Spectralis^®^ OCT (Heidelberg Engineering, Heidelberg, Germany) instruments were used to obtain all of the OCT images. The images were used for detecting recurrences of the CNV and evaluating the microstructures of the retina.

### Surgical techniques

**Fig 1:**
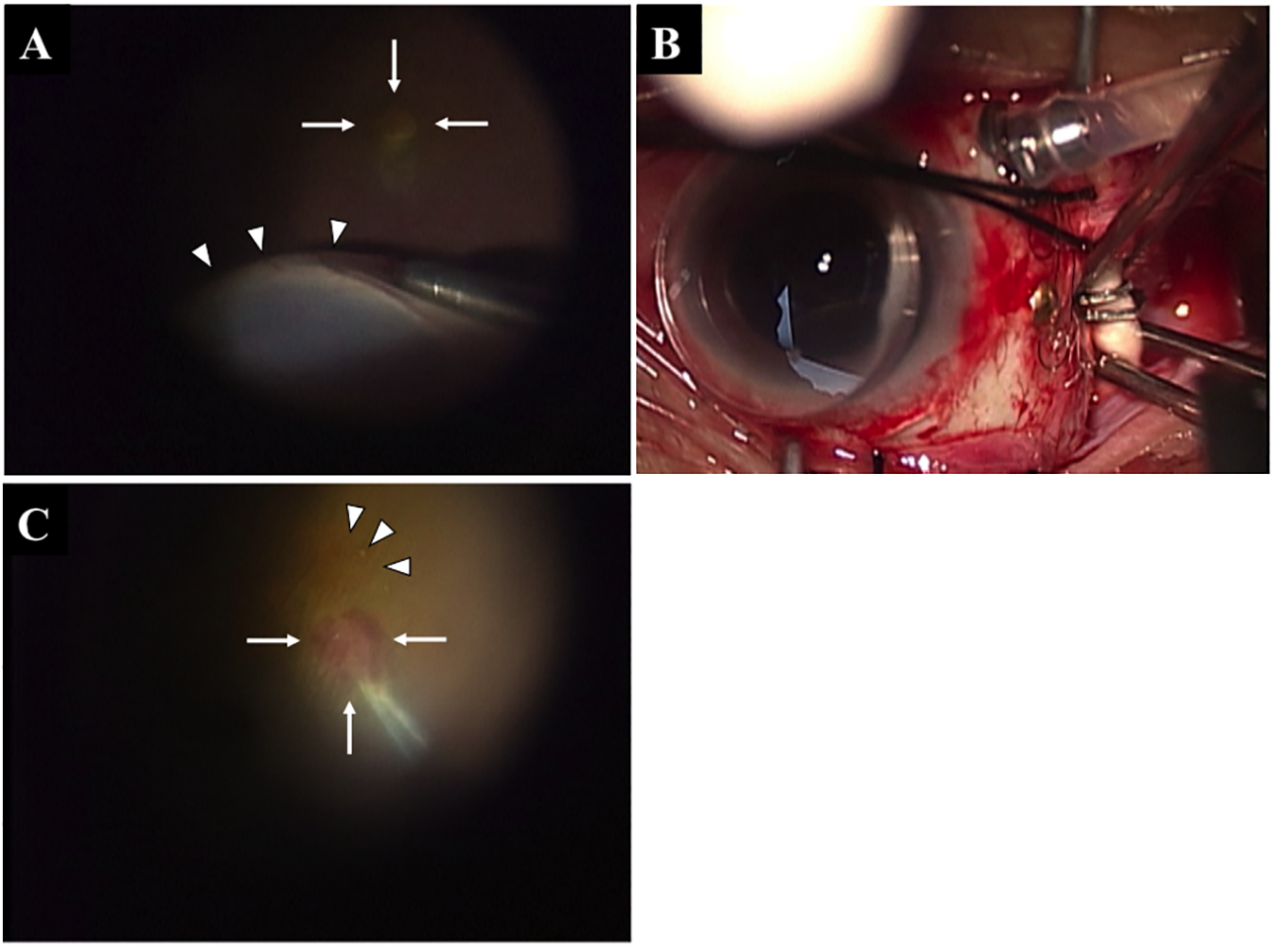
Intraoperative photographs of limited macular translocation surgery (LMT). Creating a retinal detachment (white arrowheads) using a 39-gauge injection cannula to infuse balanced salt solution (BSS) (A). White arrows show a choroidal neovascularization (CNV). Clips were placed along a diagonal line beginning 2 mm posterior to the lateral rectus muscle insertion to the superior oblique muscle insertion (B). The new fovea is shown by the white arrowheads. The CNV membrane was grasped with subretinal forceps (white arrows) and removed (C).

The procedures for the LMT surgery followed closely those described by De Juan (Fig 1). [14] After retrobulbar anesthesia, a 360° conjunctival peritomy was performed followed by placement of sutures at the insertions of the superior and lateral rectus muscles. A 3-port pars plana vitrectomy with the creation of a complete posterior vitreous detachment was then carried out. Then, a 39-gauge injection cannula (Synergetics, St Charles, MO, USA) was used to infuse balanced salt solution (BSS) into the subretinal space to detach the superior, temporal, and inferior retina. The infusion was carried out with the vitreous fluid injector module settings on an Accurus vitrectomy instrument (Alcon Laboratories, Fort Worth, TX). The retinal detachment tended to expand peripherally (Fig 1A). Air–fluid exchange was performed to hydraulically dissect the macula as the BSS moved posteriorly. Once the sensory retina was detached at the posterior pole and the temporal quadrants, the superotemporal sclera was exposed by pulling the preset sutures in the insertion sites of the superior and later rectus muscles. Chorioscleral shortening was achieved by using forceps to outfold an area of sclera to a width of 2.0 to 2.5 mm and a length of 10 mm in a diagonal direction from 2 mm posterior to the insertion of the lateral rectus muscle to the insertion of the superior oblique muscle. The outfolded sclera was then secured with titanium clips (Fig 1B). One L-sized clip (DuraClose, Tyco Healthcare Japan, Tokyo, Japan) was placed 2 mm posterior to the lateral rectus muscle insertion, and a 5 L-sized clips was placed on a diagonal line toward the superior oblique muscle insertion (Fig 1B). Finally, a partial fluid–air exchange was performed.

After the surgery, the patient’s head was positioned with the nasal side of the operated retina up for 15 to 30 minutes, then with the superonasal side up (lying on the temporal side with the head raised approximately 45 to 60 degrees with a pillow) for approximately 2 hours, upright overnight, superotemporal side up (lying on the nasal side with the head raised approximately 45 to 60 degrees) for half a day, and finally with the temporal side up until the air bubble disappeared.

Ten to 24 days after the LMT surgery, 3-port for pars plana vitrectomy was performed to remove the CNV. A fine cannula was used to make a retinotomy that was away from the new foveal center. After a subretinal injection of a small volume of BSS and separation of the neurosensory retina from the pigment epithelium, the CNV membrane was grasped with subretinal forceps and extracted through the retinotomy (Fig 1C). Then, fluid–air exchange was performed.

### Best-corrected visual acuity (BCVA)

The decimal BCVA was recorded at each visit and the acuity was converted to the logarithm of the minimal angle of resolution (logMAR) for statistical analyses. When the change of the BCVA was greater than ±0.2 logMAR units, the change was defined as an improvement or a worsening. Multiple logistic regression analysis was used to identify factors that contributed to the final BCVA.

### Statistical analyses

Independent sample *t* tests and nonparametric Mann-Whitney *U* tests were used to determine the significance of the differences in the variables. Fisher exact tests were used to compare the qualitative variables. Repeated-measure analysis of variance was used to compare differences at the different time points after the surgery. Differences with a *P* <0.05 were considered statistically significant.

## Results

### Patient demographics

**Fig 2:**
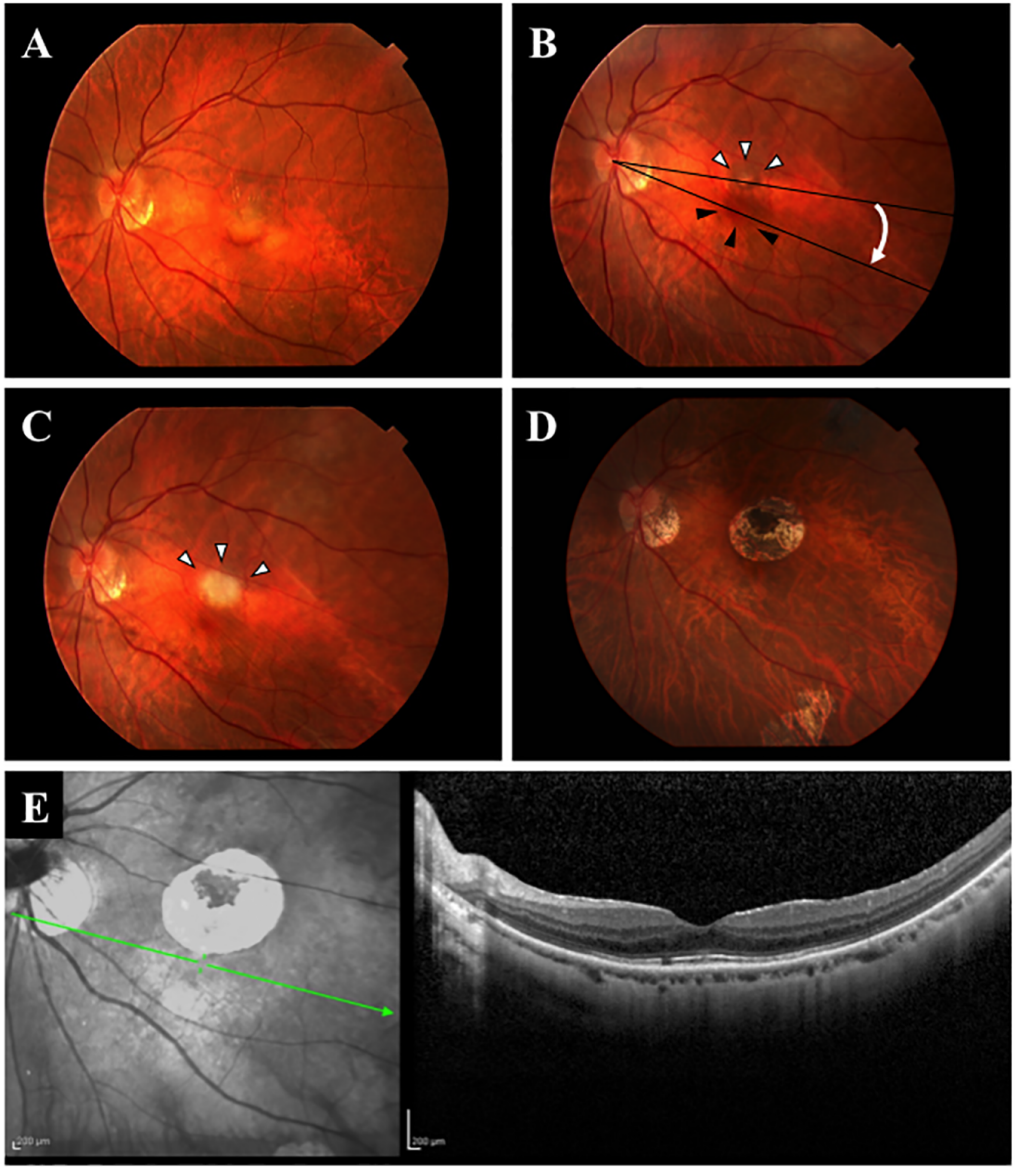
Representative eye before and after limited macular translocation surgery (LMT). Fundus photographs showing a subfoveal choroidal neovascularization (CNV) with serous detachment, and minimal subretinal hemorrhage at the macula before the surgery (A). The macula was moved inferiorly to the new healthy RPE (black arrowheads), and the CNV can be seen (white arrowheads) after the LMT. The CNV was removed 2 weeks after the LMT, black arrows point to the scar after the CNV was removed (C). An expanded atrophic area can be seen 2 years after the surgery (D). A spectral domain optical coherence tomographic image showing that the anatomic structure is normal at the fovea 6 years after surgery (E).

Twenty-two eyes of 22 consecutive Japanese patients (14 men and 8 women) underwent LMT surgery from July 2001 to November 2003. Of the 22 patients, 2 patients were excluded because the follow-up period was less than 5 years. Thus, 20 patients (13 men and 7 women) were included in this study with a mean follow-up period of 7.1 ± 1.8 years. None of the eyes had an intravitreal injection or photodynamic therapy (PDT) before the surgery. The demographic data of the patients and the treatment results are summarized in Table 1. The mean age at the time of the LMT surgery was 65.2 ± 9.8 years with a range of 41–80 years. The macula was successfully translocated in all eyes (Fig 2, Table 1). The size of CNV was 1724 ± 682 μm with a range of 650–3110 μm. At the second surgery, the CNV was removed in 16 eyes, and the CNV was photocoagulated in the other 4 eyes.

**Table 1:** Characteristics of the patients with age-related macular degeneration.

No	Age (year)	Gender		Visual acuity				Foveal displacement (μm)	CNV removal	Complication	Place of recurrence	Time to Recurrence (months)
				Initial	1 year	5 year	Last					
1	69	M		20/60	20/25	20/50	20/50	1280	+			
2	74	F		20/500	20/500	20/500	20/500	1080	+	Recurrence	Subfovea	8
3	56	F		20/150	20/40	20/30	20/50	741	+	MH, Recurrence	Juxtafovea	4
4	77	F		20/100	20/200	20/400	20/1000	621	+	Recurrence	Juxtafovea	9
5	68	M		20/500	20/25	20/20	20/20	771	-			
6	76	M		20/250	20/150	20/400	20/500	868	-	Recurrence	Subfovea	2
7	56	F		20/300	20/400	20/150	20/200	1820	-			
8	57	M		20/100	20/60	20/100	20/40	1537	+			
9	75	M		20/300	20/150	20/80	20/80	1530	+			
10	55	M		20/50	20/150	20/80	20/150	1542	+	Recurrence	Subfovea	2
11	60	F		20/300	20/150	20/80	20/100	2040	+	Recurrence	Juxtafovea	12
12	67	M		20/60	20/100	20/60	20/60	1080	+			
13	72	M		20/250	20/200	20/300	20/200	1440	+			
14	80	M		20/100	20/25	20/40	20/40	1920	+			
15	74	M		20/60	20/100	20/60	20/60	1220	-			
16	60	F		20/100	20/100	20/50	20/50	1350	+			
17	60	F		20/60	20/25	20/25	20/25	1248	+			
18	65	M		20/200	20/40	20/25	20/25	1180	+			
19	41	M		20/40	20/30	20/40	20/40	1671	+	Recurrence	Juxtafovea	9
20	61	M		20/100	20/60	20/80	20/60	1200	+		
	65.2±9.8			20/135	20/77	20/80	20/89	1332±393				6.6 ±3.9

CNV: choroid neovascularization, PC: photocoagulation, MH: macular hole.

### Foveal displacements and recurrences of CNV

The mean foveal displacement was 1332 ± 393 μm with a range of 621 to 2040 μm. An insufficient displacement, i.e., the displaced fovea still remained on the CNV after surgery, occurred in 4 eyes, and these 4 eyes had a recurrence of the CNV. A CNV developed at the new fovea in 7 eyes (subfovea in 3 eyes, juxtafovea 4 eyes) in the first year after surgery (Table 1). These were treated with radiation initially because PDT was not permitted in Japan at that time. The structure of retina at the fovea on the new RPE appeared normal in the OCT images obtained at least 5 years after the surgery in the eyes without a recurrence of the CNV.

The intra- and postoperative complications included retinal tear(s) in three eyes (15%) and a MH in one eye (5%) (Table 2). There were no cases of hemorrhages, proliferative vitreoretinopathy, and ocular penetrations directly associated with the clipping. None of the patients required strabismus surgery after the LMT surgery and one patient was treated with low power prism for diplopia.

**Table 2:** Complications.

Complication	Number of eyes of 20 eyes
Insufficient displacement	4
Recurrence	7
Retinal tear	3
Macular hole	1
Diplopia	1

### BCVA after LMT

**Fig 3:**
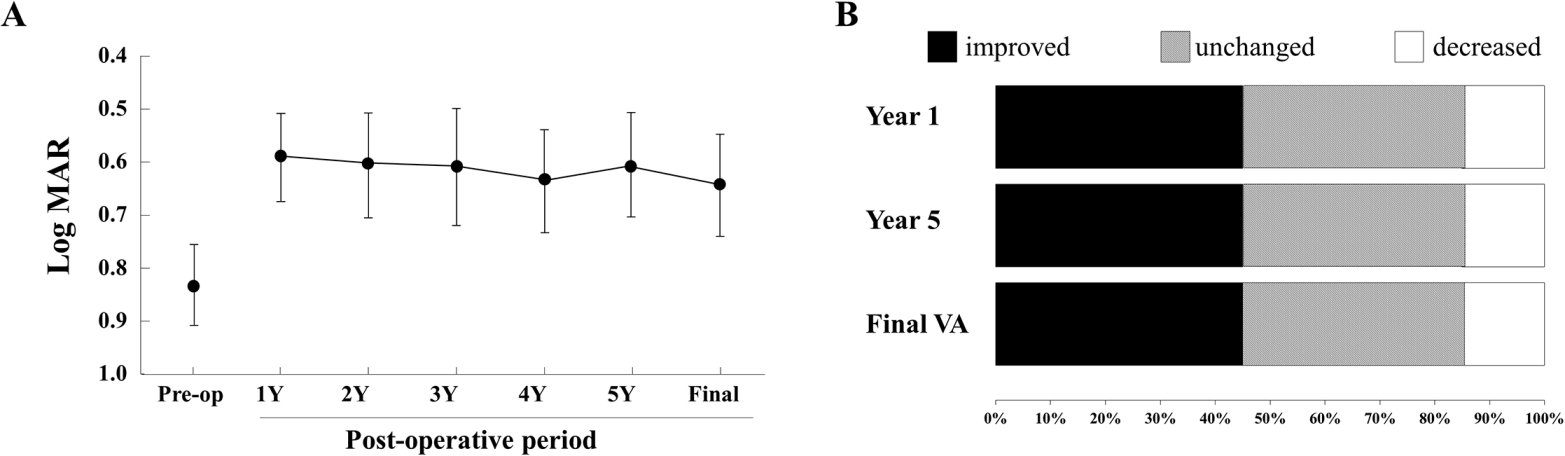
Mean best-corrected visual acuity (BCVA) ± standard error of the means (± SEM) before and at 1, 2, 3, 4, 5 years and final visit following the LMT surgery. The BCVA was significantly improved at 1 year after surgery (*P* = 0.015), and the improved BCVA was maintained for at least 5 years after the surgery. In comparison with the preoperative BCVA, the BCVAs at all of the 3 postoperative time points (Year 1, Year 5, and the final examination) are improved by 2 or more lines of the visual acuity chart in 9 of the 20 eyes (45%), no change in 8 (40%), and decreased in 3 eyes (15%).

The mean preoperative BCVA was 20/135 (0.83 ± 0.33 logMAR units) with a range of 20/50 to 20/500 (Table 1). The changes in the mean BCVA are shown in Fig 3. The mean BCVA at 1 year after surgery was 20/77 (0.59 ± 0.37 logMAR units) which was significantly better than that before surgery (*P* = 0.015). In addition, the BCVA did not worsen significantly thereafter and was 20/77 (0.59 ± 0.41 logMAR units) at the final examination.

**Fig 4:**
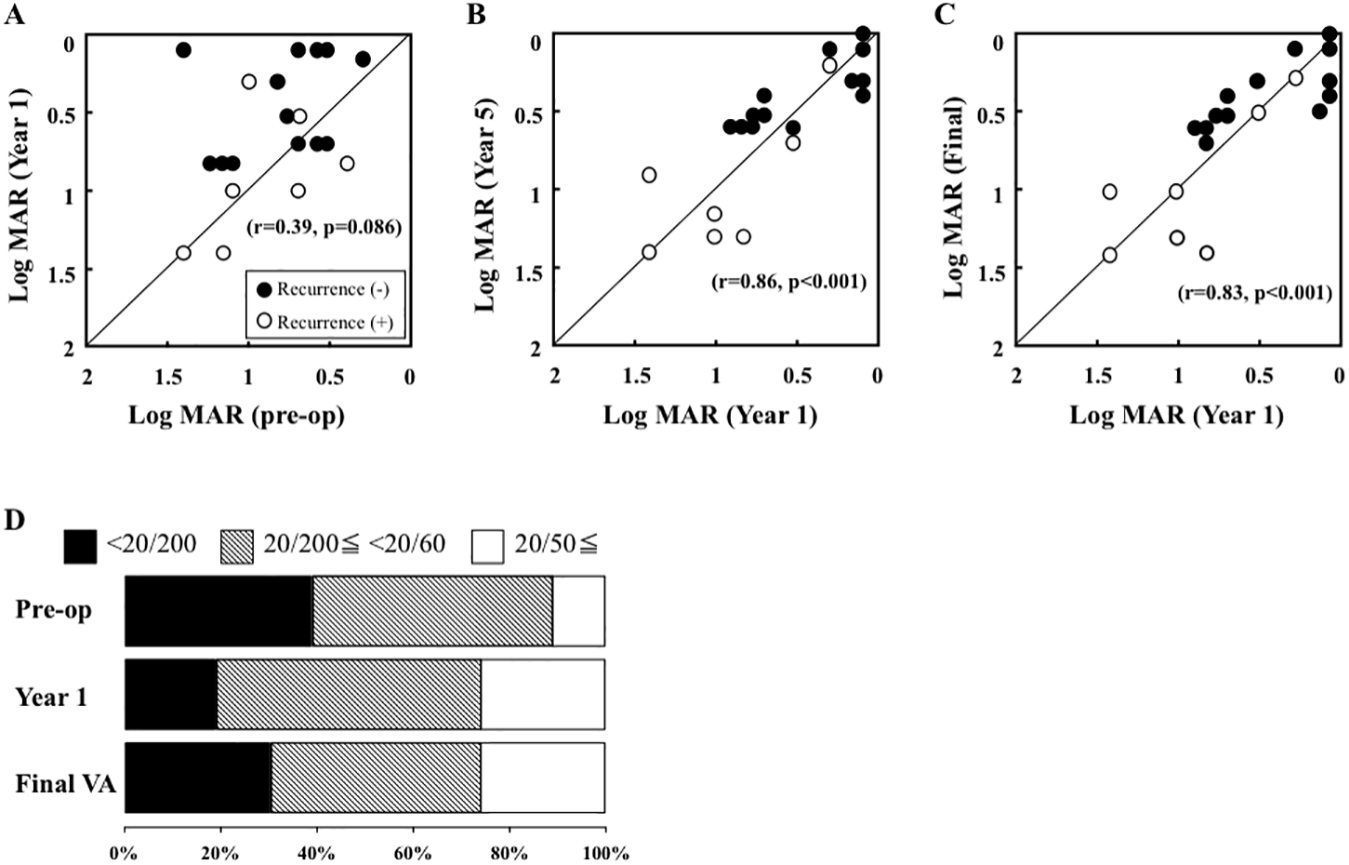
**Scatter plots of the BCVA before and 1 year after surgery (A), and the BCVA 1 year after surgery and 5 year after surgery (B) and at the final examination (C) are shown.** The BCVAs are plotted in logarithm of the minimum angle of resolution (logMAR) units. The BCVA of 13 eyes without a recurrence (filled circle) and 7 eyes with a recurrence (open circle). The mean BCVA at 1 year after surgery is not significantly correlated with that before surgery, but is significantly correlated with that at 5 year after the surgery (r = 0.86, *P* <0.001) and the final follow-up examination (r = 0.83, *P* <0.001). The BCVA at 1 year after surgery was 20/200 or worse in 4 eyes (20%), 20/200 to 20/60 in 11 eyes (55%), and 20/50 or better in 5 eyes (25%). The final BCVA was 20/200 or worse in 6 eyes (30%), 20/200 to 20/60 in 9 eyes (45%), and 20/50 or better in 5 eyes (25%) (D).

The postoperative BCVA improved by 2 or more lines in 9 eyes (45%), no change in 8 eyes (40%), and a decrease by 2 or more lines in 3 eyes (15%) at 1 year after surgery. The 3 eyes with a decrease by 2 or more lines was due to a recurrence of a CNV, and the BCVA at 1 year after surgery was not significantly correlated with that before the surgery (Fig 4). However, the BCVA at 5 year after the surgery (r = 0.86, *P* <0.001) and at the final follow-up examination (r = 0.83, *P* <0.001) were significantly correlated with the BCVA at 1 year after the surgery. The BCVA in the 7 eyes with a recurrence remained lower than that of the 13 eyes without a recurrence throughout the follow-up period. The BCVA at 1 year after surgery was 20/200 or worse in 4 eyes (20%), 20/200 to 20/60 in 11 eyes (55%), and 20/50 or better in 5 eyes (25%), and the final BCVA was 20/200 or worse in 6 eyes (30%), 20/200 to 20/60 in 9 eyes (45%), and 20/50 or better in 5 eyes (25%).

The result of the multiple linear regression analysis for the final BCVA are shown in Table 3. The BCVA at 1 year after surgery (*P* <0.001), the recurrence of a CNV (*P* = 0.001), and age (*P* = 0.022) contributed significantly to the final BCVA, but the sex, size of the CNV, displacement of the CNV, and preoperative BCVA were not significantly associated with the final BCVA (Table 3).

**Table 3:** Multiple regression analysis of factors contributing to the final visual acuity.

Explanatory variables	coefficients	*t*-value	*p*-value
Visual acuity at Year 1	0.697	7.011	<0.001
Recurrence (yes/no)	-0.399	-4.066	0.001
Age	0.251	-2.541	0.002
Gender (M/F)	-0.165	-1.531	0.147
Size of CNV	-0.131	-1.315	0.208
Displacement of CNV	0.123	1.137	0.273
Preoperative visual acuity	0.002	0.240	0.981

CNV = choroidal neovascularization.

## Discussion

Our findings showed that the BCVA of eyes with AMD was significantly improved at 1 year after the LMT surgery, and the improved BCVA was maintained for at least 5 years after the surgery. The BCVA at 1 year after surgery was not significantly correlated with that before the surgery but was significantly correlated with the BCVA at the final examination. In addition, multiple linear regression analyses showed that the final BCVA was also significantly correlated with recurrences of the CNV and age. To the best of our knowledge, this is the first report describing the outcomes in LMT surgery followed for more than 5 years.

There have been many studies that reported that the postoperative vision improved after LMT surgery. For example, Fujii et al.[16] and Lewis[22] reported that the BCVA improved by 2 or more lines in 39.5% and 44%, respectively, of their cases. Chang et al reported that the mean BCVA improved by 0.19 logMAR units at 1 year after the LMT surgery.[20] These findings are in good agreement with our results in which the postoperative vision improved by 2 or more lines in 45%, and the mean improvement in BCVA was by 0.24 logMAR at 1 year after the surgery. These results indicate that the impaired function of the sensory retina of the fovea can recover when the retina is translocated onto healthy RPE.

Although the earlier studies reported favorable outcomes, the follow-up period was approximately 1 year after the LMT surgery. Kamei et al. reported that the mean BCVA at 2 years after LMT surgery was not significantly different from the preoperative BCVA. A failure of an improvement in the BCVA was attributed to the enlargement of the CNV.[18] The 7 eyes with recurrences of the CNV in our study occurred within the first year, and the mean BCVA at 5 years after the LMT surgery and the final examination was not significantly worse than that at 1 year after the surgery. The difference between Kamei et al and our study is that we removed the CNV immediately after the LMT surgery in most of the cases, and they removed the CNV only after an enlargement of the CNV. In our patients, the decrease of 2 or more lines in the standard visual acuity chart in 3 eyes was due to a recurrence of a CNV. In addition, the multiple regression analyses showed that the recurrence of CNV contributed to the final vision. Taken together, the prevention of a CNV recurrence is very important for achieving good vision for a long period after the LMT surgery.

When we performed the LMT surgery, we had no other options to manage the CNV except to remove it. At present, we have other options, e.g., PDT or intravitereal injection of anti-VEGF agents, to prevent recurrences.

Takeuchi et al report that the BCVA of eyes with larger CNVs improved significantly after full macular translocation surgery at 1 year, and the BCVA did not change significantly for more than 5 years after the full macular translocation surgery[23] as was found in our patients. In addition, our study showed that the BCVA at 1 year after surgery was significantly correlated with that 5 year after surgery and at the final follow-up examination. Together, these results suggest that if good vision is achieved at 1 year after a translocation surgery, the improved BCVA will be maintained for at least 5 years after the surgery. In addition, we found that the structure of the sensory retina at the fovea on the new RPE appears to be normal for a long time in the eyes without a recurrence of CNV in most of the cases. Thus, macular translocation can move the macula from the underlying damaged RPE to an area of healthier RPE, and the BCVA and structure of the retina will recover and be maintained for at least 5 years.

Takeuchi et al reported that the 5 of 35 eyes (14%) with AMD treated by 360 full macular translocation had a recurrence of CNV which is much fewer than our results. In eyes with 360 full macular translocation, the CNV can be observed directly after making the retinotomy and flipping the retina during surgery. On the other hand, it is possible to see that part of the CNV remaining when removing the CNV from a small retinotomy during LMT because the CNV can be seen though the sensory retina. The failure to remove all of the CNV may be the cause recurrences from the residual CNV.

One of the main drawbacks of LMT surgery is that the degree of foveal displacement is unpredictable. Kamei et al. compared 3 techniques used in LMT surgery and concluded that radial shortening was the preferable method for obtaining a greater displacement.[22, 24] The use of neurosurgical clips to secure the outfolded sclera-retina should shorten the eye wall more and produced more redundant retina than does the suture infolding technique. The mean displacement of the fovea in their modified technique was 1576 μm,[18] which was greater than that of other reports.[16, 22] However, they stated that the results of displacement still varied. We used the technique which Kamei et al introduced,^18^ and the mean displacement of the fovea was 1332 μm which is greater than that reported earlier.[16, 22, 25, 26] In cases with insufficient displacement, the fovea would remain on the CNV after the surgery. This occurred in 20% of the eyes in our study and was related to the recurrence of CNV. The rate of recurrences was similar to that reported by Mateo et al.[27] and by Fujii et al.[28] who reported insufficient foveal displacement in 20% and 45% of their patients, respectively.

None of the patients required strabismus surgery after the LMT, and one patient was treated with 20 prism diopters for diplopia. This is the advantage of LMT surgery in having fewer patients requiring strabismus surgery. After 360° retinotomy and full macular translocation, the rate of diplopia was much higher than after LMT because of the larger foveal displacements.

Our study has limitations. First, this study was a nonrandomized and retrospective study with a small sample size. However, it is difficult to increase the sample size because the number of eyes that require LMT surgery is limited, and intravitreal injections of anti-VEGF agent is the preferred treatment at present. Second, LMT surgery significantly improved the BCVA, but the BCVA after LMT is lower and the complication rate is higher in comparison to intravitreal anti-VEGF treatment at 5 years. Third, the CNV was not surgically excised in all of the cases because the foveal displacement was not sufficient and part of the CNV was still on the new fovea.

In conclusion, LMT surgery can improve the BCVA at 1 year after surgery and the improved BCVA is maintained for more than 5 years after the surgery. The results suggest that the sensory retina can function for at least 5 years on the new healthy RPE. In the future, cost-effectiveness analyses and combination/rescue therapies may be developed based on our results. The ability to maintain good retinal function on the new RPE for a long period which our results showed is important for future treatments of CNVs such as the transplantation of RPE cells and stem cells.

## Supporting information

S1 File Dataset. (XLSX)
